# Direct Visualization of Fungal Burden in Filamentous Fungus-Infected Silkworms

**DOI:** 10.3390/jof7020136

**Published:** 2021-02-13

**Authors:** Yidong Yu, Ann-Katrin Wolf, Sina Thusek, Thorsten Heinekamp, Michael Bromley, Sven Krappmann, Ulrich Terpitz, Kerstin Voigt, Axel A. Brakhage, Andreas Beilhack

**Affiliations:** 1 Interdisciplinary Center for Clinical Research Laboratory, Department of Internal Medicine II, Würzburg University Hospital, 97080 Würzburg, Germany; Wolf_A7@ukw.de (A.-K.W.); Thusek_S@ukw.de (S.T.); 2 Research Center for Infectious Diseases, 97080 Würzburg, Germany; 3 Department of Molecular and Applied Microbiology, Leibniz Institute for Natural Product Research and Infection Biology—Hans Knöll Institute, 07745 Jena, Germany; Thorsten.Heinekamp@hki-jena.de (T.H.); Axel.Brakhage@hki-jena.de (A.A.B.); 4 Manchester Fungal Infection Group, University of Manchester, Manchester M13 9PL, UK; mike.bromley@manchester.ac.uk; 5 Institute for Clinical Microbiology, Immunology and Hygiene, Erlangen University Hospital, 91054 Erlangen, Germany; Sven.Krappmann@uk-erlangen.de; 6 Erlangen Center of Infection Research, Friedrich-Alexander University of Erlangen-Nürnberg, 91054 Erlangen, Germany; 7 Department of Biotechnology and Biophysics, Theodor-Boveri-Institute, Biocenter, University of Würzburg, 97074 Würzburg, Germany; ulrich.terpitz@uni-wuerzburg.de; 8 Jena Microbial Resource Collection, Leibniz Institute for Natural Product Research and Infection Biology—Hans Knöll Institute, 07745 Jena, Germany; kerstin.voigt@leibniz-hki.de; 9 Institute of Microbiology, University of Jena, 07743 Jena, Germany

**Keywords:** fungal infection model, calcofluor white staining, *Aspergillus*, *Lichtheimia*, silkworm

## Abstract

Invasive fungal infections (IFIs) are difficult to diagnose and to treat and, despite several available antifungal drugs, cause high mortality rates. In the past decades, the incidence of IFIs has continuously increased. More recently, SARS-CoV-2-associated lethal IFIs have been reported worldwide in critically ill patients. Combating IFIs requires a more profound understanding of fungal pathogenicity to facilitate the development of novel antifungal strategies. Animal models are indispensable for studying fungal infections and to develop new antifungals. However, using mammalian animal models faces various hurdles including ethical issues and high costs, which makes large-scale infection experiments extremely challenging. To overcome these limitations, we optimized an invertebrate model and introduced a simple calcofluor white (CW) staining protocol to macroscopically and microscopically monitor disease progression in silkworms (*Bombyx mori*) infected with the human pathogenic filamentous fungi *Aspergillus fumigatus* and *Lichtheimia corymbifera*. This advanced silkworm *A. fumigatus* infection model could validate knockout mutants with either attenuated, strongly attenuated or unchanged virulence. Finally, CW staining allowed us to efficiently visualize antifungal treatment outcomes in infected silkworms. Conclusively, we here present a powerful animal model combined with a straightforward staining protocol to expedite large-scale in vivo research of fungal pathogenicity and to investigate novel antifungal candidates.

## 1. Introduction

In the past decades, invasive fungal infections (IFIs) have been continuously rising, particularly among immunocompromised individuals [1,2,3]. More recently, IFIs, and especially those caused by *Aspergillus* species, were found worldwide in critically ill patients admitted to the intensive care unit (ICU) following influenza or SARS-CoV-2 infections [4,5,6,7,8,9,10]. To date, IFIs are still associated with high mortality rates due to diagnostic and therapeutic limitations [11,12]. Fighting against IFIs requires a deeper and more comprehensive understanding of fungal virulence determinants, which helps to develop novel antifungal strategies.

So far, animal models are still indispensable for studying fungal pathogenicity as well as for the development of new antifungals. The use of mammals, e.g., mice and rats, faces various challenges such as increasing regulatory hurdles, ethical concerns, and high costs that almost preclude large-scale experiments. To overcome these challenges, several invertebrate infection models were proposed as a cost- and time-effective replacement for mammalian hosts with respect to large-scale, preliminary in vivo studies. Present major invertebrate models include the fruit fly (*Drosophila melanogaster*), a nematode (*Caenorhabditis elegans*), larvae of the greater wax moth (*Galleria mellonella*), the domestic silk moth (*Bombyx mori*), or the tobacco hornworm moth (*Manduca sexta*) [13,14,15].

Compared to other major invertebrate models, silkworms (larvae of the silk moth) have several advantages [14,16,17,18]: 

(1) Easy to rear in laboratories with a very low risk of biohazard; 

(2) Larger body size (~5 cm), which allows easy handling and particularly injections; 

(3) Larger injection volume (up to 50 µL), which enables accurate dose titrations; 

(4) Feasibility of both intra-hemolymph and intra-midgut injections, which mimic intravenous and oral administrations in mammals, respectively; 

(5) Comparable therapeutic effects, pharmacokinetic parameters, and toxicity of antimicrobial drugs in silkworms and mammals. 

Silkworm infection models have been established for various human fungal pathogens [14,19], including yeasts and filamentous fungi, such as *Candida* species, *Cryptococcus neoformans*, *Trichosporon asahii*, *Aspergillus fumigatus*, *Rhizopus oryzae*, and several dermatophytes. A silkworm infection model of *Candida albicans* has been applied for screening of null mutants to identify virulence-related genes [20]. Furthermore, a novel antifungal agent named VL-2397 (former: ASP2397) that eventually entered clinical trials was discovered through screening of a natural product library in a silkworm infection model of *A. fumigatus* [21,22]. Taken together, silkworm infection models can not only be used for the identification of infection-related fungal genes, but also for the discovery of potential antifungal agents.

Although silkworm infection models have been established for different fungal pathogens, microscopical visualization of fungal burden in silkworms so far relies on recombinant fungal strains that express fluorescent proteins [19,23] or on classical histological staining procedures such as Grocott-Gomori’s methenamine silver stain or periodic acid-Schiff stain in tissues, which are time-consuming due to fixation, sectioning, dehydration, and staining steps [24,25].

In this study, we introduced a simple calcofluor white (CW) staining protocol for visualization of fungal growth in freshly extracted silkworm hemolymph and organs. Given that CW has been widely used as a fluorescent dye to visualize the fungal cell wall [26], we included two distinct filamentous fungal pathogens, the ascomycete *A. fumigatus* and the mucoromycete *Lichtheimia corymbifera*, to demonstrate the general applicability of our staining protocol. With the help of CW staining, we could not only compare fungal burden in silkworms infected with distinct fungal mutants, but also monitor microscopically the outcome of antifungal treatment, as demonstrated by using two conventional antifungals, voriconazole and amphotericin B.

## 2. Materials and Methods

### 2.1. Strains, Media, and Culture Conditions

*Aspergillus fumigatus* strains used in this study are listed in Table S1 in Supplementary Material. All the strains were grown at 37 °C for 3 days on a solid *Aspergillus* minimal medium (AMM) containing 1% glucose as a sole carbon source, 1× AspA solution (70 mM NaNO_3_, 7 mM KCl, 11 mM KH_2_PO_4_, pH 5.5), 2 mM MgSO_4_, and 1× trace elements solution [27]. Recombinant fungal strains possessing the *hph* and *ptrA* genetic markers [28,29] were grown with an additional supply of 100 µg/mL hygromycin B and 0.05 µg/mL pyrithiamine, respectively.

Fresh conidiospores were harvested with 0.9% NaCl/0.01% Tween 20 (NaCl-Tween; both NaCl and Tween 20 were purchased from Carl Roth, Karlsruhe, Germany) from AMM plates, filtered through a 40 µm cell strainer (Corning Incorporated, Durham, NC, USA), and centrifuged at 3750× *g*, 4 °C for 10 min. After removing the supernatant, the pellet was resuspended in NaCl-Tween and stored at 4 °C until use (up to 7 days).

The *Lichtheimia corymbifera* reference strain JMRC:FSU:09682 was used in this study [30]. Cultivation was performed on a solid KK1 medium at 30 °C for 7 days as previously described [31]. Fresh sporangiospores were harvested with a phosphate buffered saline solution (PBS, pH 7.4; Carl Roth, Karlsruhe, Germany) from KK1 plates, filtered through a 40 µm cell strainer, and washed twice by centrifugation at 2000× *g*, room temperature for 5 min. After removing the supernatant, the pellet was resuspended in NaCl-Tween and stored at 4 °C until use (up to 7 days).

### 2.2. Chemicals

Calcofluor white M2R (fluorescent brightener 28), l-glutathione reduced (GSH), voriconazole, and amphotericin B solution (250 µg/mL in deionized water) were purchased from Sigma-Aldrich (St. Louis, MO, USA). Calcofluor white M2R was prepared as a 0.35% (*w*/*v*) working solution in distilled water with a small amount of 10 N sodium hydroxide (NaOH; 2–3 drops/10 mL water) to facilitate the dissolving (hereafter referred to as a CW staining solution). The GSH working solution (200 mM in 0.9% NaCl) was freshly prepared prior to use. The voriconazole stock solution was prepared at a concentration of 5 mg/mL in dimethyl sulfoxide (DMSO). Both voriconazole and amphotericin B stock solutions were further diluted in 0.9% NaCl prior to injection.

### 2.3. Animal Model of Infection

Four-way polyhybrid silkworm eggs (129 × 127) × (126 × 125) and two types of dry fodder (with and without preservatives) were purchased from the Council for Agricultural Research and Economics—Research Centre for Agriculture and Environment (CREA-AA, Padova, Italy). A simplified silkworm rearing protocol (laboratory version) is depicted in Figure S1. Silkworm diet was prepared according to the manufacturer’s protocol. Briefly, silkworm larvae were fed with a 25% artificial mulberry diet [32] with preservatives until they entered the fifth instar. On the first and second days of the fifth instar, silkworms were fed with a 25% artificial mulberry diet without preservatives. Silkworms were used for infection experiments on the third day of the fifth instar. Of note, for short-term experiments (visualization at time point 8 or 16 h post infection), silkworms were fasted at 25 °C overnight prior to infection (on the fourth day of the fifth instar).

Fungal infection was performed in a biosafety cabinet. A 50 µL suspension of *A. fumigatus* or *L. corymbifera* spores in NaCl-Tween was injected into the hemolymph through the dorsal epidermis of the silkworm at the fifth segment [14] using a 1 mL syringe with a 30-gauge needle (ø 0.30 × 12 mm; B. Braun). In general, an infectious dose of *A. fumigatus* strains was 2 × 10^5^ spores/50 µL or 2 × 10^4^ spores/50 µL of *L. corymbifera*. Of note, lower infectious doses were used for experiments involving the following antifungal treatment: 1 × 10^5^ spores/50 µL (*A. fumigatus* A1160p+) or 1 × 10^4^ spores/50 µL (*L. corymbifera* JMRC:FSU:09682). Unless otherwise indicated, the typical group size is 10–20 silkworms/group in each experiment. After infection, silkworms were incubated at 34 °C without feeding and their survival was monitored.

### 2.4. Visualization of Fungal Growth in Silkworm Hemolymph and Organs

For visualization of fungal growth in the hemolymph, spore suspension (in NaCl-Tween) at the indicated concentration was stained with CW shortly before intra-hemolymph administration, i.e., 1.67 µL of the CW staining solution was added to 50 µL of the spore suspension (1:30 ratio). Silkworms were anesthetized on ice for 40 s, followed by cutting one of the abdominal legs to gently drain the hemolymph. The GSH working solution was immediately added to the hemolymph at a final concentration of 5 mM to prevent melanization [33,34]. In addition, 40–50 µL of the drained hemolymph was carefully loaded into a six channel μ-slide (ibidi) shortly before microscopy. Notably, hemolymph extraction at late time points post infection (>30 h) was difficult, given that (1) silkworms at their fifth instar naturally release water in preparation for spinning cocoons [35]; (2) silkworms were fasted post infection in this study. Thus, we could monitor fungal growth in the hemolymph when the incubation time did not exceed 30 h.

**Figure 1:**
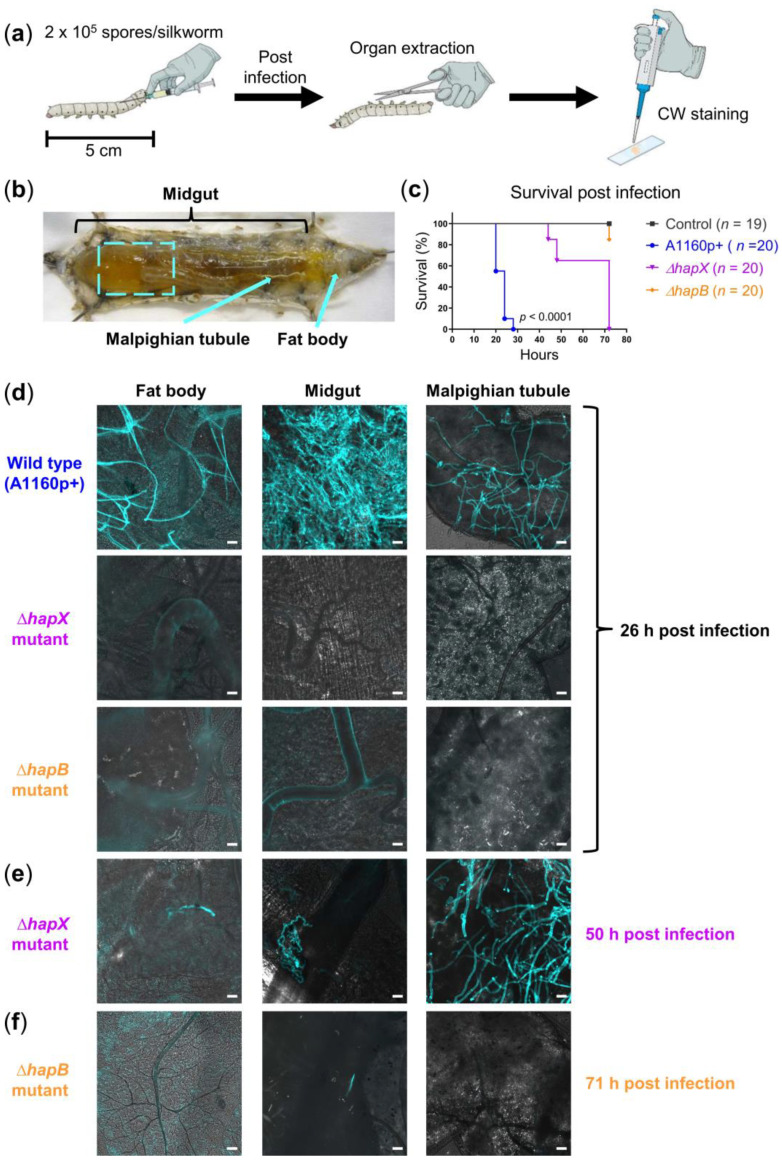
Comparison of fungal burden in silkworms infected with different *A. fumigatus* strains. (**a**) Illustration of experimental setup; CW: Calcofluor white; (**b**) anatomy of silkworm: Dashed square indicates the area of midgut taken for microscopy; arrows indicate the Malpighian tubule, and the area where fat body was extracted; (**c**) survival of silkworms infected with 2 × 10^5^ spores of the wild type (WT) strain A1160p+, *ΔhapX* or *ΔhapB* mutant; silkworms in the control group were injected with 50 µL NaCl-Tween; *n*: Number of silkworms per group; Kaplan-Meier survival curves were compared using the log-rank test; pairwise comparisons were performed between the A1160p+ and *∆hapX* groups, as well as between the A1160p+ and *∆hapB* groups; both comparisons show *p*-values < 0.0001; (**d**) merged images (calcofluor white in turquoise + brightfield) show fungal burden in different organs of WT-infected silkworms at 26 h post infection, while hyphae of the *ΔhapX* and *ΔhapB* mutants were detectable in the organ(s) at 50 h (**e**) and 71 h (**f**) post infection, respectively; scale bars represent 20 µm.

For organ extraction, silkworms were anesthetized under ice for 10 min. Afterwards, the head and tail of the larva (laying on its dorsal side) were fixed at the longest-possible distance by needles on a styrofoam dissection plate. Subsequently, the abdominal epidermis was carefully cut along the midline with dissection scissors, followed by fixation of cut epidermis on both sides with needles to expose the organs underneath. As shown in Figure 1b, fat body, midgut, and one Malpighian tubule of the silkworm were extracted, placed on microscope slides with 15 µL 0.9% NaCl and 2 µL CW staining solution, and pressed flat by cover slips. Quick-dry nail polish was used to seal the cover slips on the microscope slides. Samples were either immediately examined under a microscope or kept in the dark at 4 and −20 °C for short-term and long-term storage, respectively. All the samples were examined using ultraviolet light (for CW: Optimum excitation wavelength 347 nm; peak emission wavelength 450 nm) and transmitted light (for brightfield) under an inverted confocal microscope with a 20× objective (LSM 780, Zeiss, Oberkochen, Germany).

### 2.5. Antifungal Treatment

The concentration range of voriconazole and amphotericin B (1, 3.2 or 10 µg/silkworm/day) used in this work was chosen depending on data from a previous study [21]. The injection volumes of voriconazole and amphotericin B were 20 and 40 µL, respectively. Unless otherwise indicated, the typical group size is 10–15 silkworms/group in each experiment.

For silkworms infected with *A. fumigatus*, the antifungal treatment was performed twice at time point 1 and 24 h post infection by injection into the hemolymph as described above, unless otherwise indicated.

For silkworms infected with *L. corymbifera*, 20 µL 0.9% NaCl containing 10 µg of voriconazole or 40 µL 0.9% NaCl containing 10 µg of amphotericin B was injected into the hemolymph at time point 1 h post infection.

### 2.6. Statistical Analysis

Kaplan-Meier survival curves were compared using the log-rank test (pairwise comparisons) with the GraphPad Prism software (v.9.0.0) and *p*-values smaller than 0.05 were deemed statistically significant.

## 3. Results

### 3.1. Optimization of a Silkworm Aspergillus Fumigatus Infection Model to Generate a Robust and Fast In Vivo Read-Out System

The initial silkworm *A. fumigatus* infection model established by Nakamura et al. [21] relies on a rather high infectious dose (1.5 × 10^6^ spores of the clinical isolate FP1305), which results in the death of silkworms within 43–46 h at 30 °C post infection. However, 30 °C is lower than 37 °C, which is the human body temperature as well as the optimal growth temperature of *A. fumigatus*. Since silkworms are ectothermic, we first asked whether it is possible to increase the incubation temperature post infection, which could accelerate the growth of *A. fumigatus* in vivo and thus increase virulence to shorten the incubation time. In a pilot experiment at elevated temperatures, 50% of uninfected silkworms incubated at 37 °C died within 72 h, while all silkworms incubated at 34 °C were still alive at this time point (in fact, 80% of them could survive for at least 120 h; Figure S2a). Thus, we chose 34 °C as the standard incubation temperature for infection experiments, since it is better tolerated by most of the silkworms and closer to the optimal 37 °C.

Upon increasing the incubation temperature to 34 °C, we asked whether a lower infectious dose of *A. fumigatus* might already be lethal within a short period of time. Indeed, 2 × 10^5^ spores of the strain A1160p+ [36] were able to kill the silkworms within 20–28 h at 34 °C (Figure 1c), which shortened the incubation time approximately by half compared to the original model (43–46 h). Thus, we chose this infectious dose for the following experiments with *A. fumigatus* unless otherwise indicated.

### 3.2. Calcofluor White (CW) Staining Reveals Fungal Burden in Infected Silkworms

**Figure 2:**
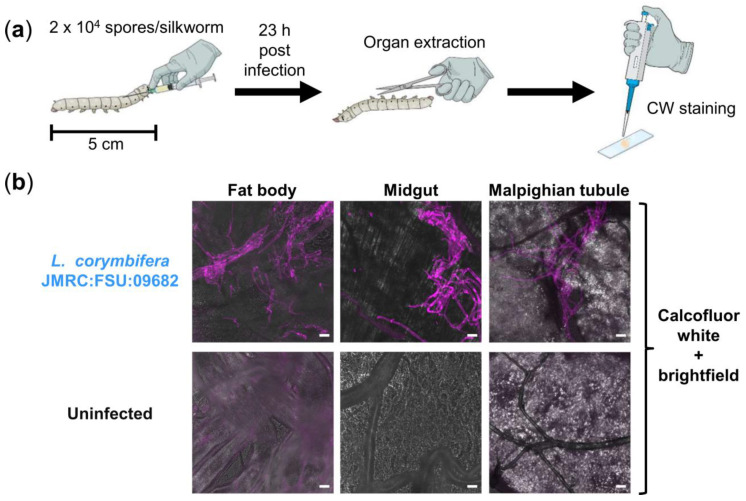
Visualization of fungal burden in silkworms infected with *L. corymbifera*. (**a**) Illustration of experimental setup; CW: Calcofluor white; (**b**) merged images (calcofluor white in violet + brightfield) show fungal burden in different organs of infected silkworms at 23 h post infection, compared to an uninfected control; scale bars represent 20 µm.

Traditionally, studying fungal burden in infected silkworms was limited to the usage of transgenic fungal mutants expressing fluorescent proteins [19,23], or to the application of time-consuming histological staining procedures [24,25]. To circumvent these limitations, we established a simple staining protocol using a common fluorescent dye, CW, to stain the cell wall of fungal hyphae in freshly extracted organs of *A. fumigatus*-infected silkworms, as demonstrated in Figure 1. To prove the general practical benefit of CW staining for diverse filamentous fungal pathogens, we applied the same staining protocol also for visualizing *L. corymbifera* (Figure 2), which can cause fulminant infections in patients with impaired immunity [37]. Briefly, our staining protocol simply required the addition of 15 µL 0.9% NaCl and 2 µL CW staining solution onto a microscope slide, followed by immersing the extracted organ. Therefore, samples were ready for microscopical examination without delay by sample processing. CW staining revealed bright signals of extensive hyphal/mycelial growth in the organs of *A. fumigatus*- and *L. corymbifera*-infected silkworms, respectively, in the final infection phase (1–2 h before death, Figure 1d and Figure 2b). We examined the fat body, midgut, and Malpighian tubule of silkworms. The fat body of silkworms is involved in energy storage, metabolism, and partial detoxification [38,39]. The midgut of silkworms is considered equivalent to the intestine of mammals [18], but recently it has also been demonstrated to play a central role in detoxification/metabolism of exogenous compounds [39]. The Malpighian tubules of silkworms correspond to the mammalian kidneys [18]. Notably, depending on the amount of administered fungal spores we observed a corresponding fungal burden. However, CW signals reached saturation when silkworms had been infected with 2 × 10^5^ spores of *L. corymbifera*. To avoid too bright signals due to the extensive mycelial growth, we adjusted the infectious dose to 2 × 10^4^ *L. corymbifera* spores, in this case we could still observe comparable fungal growth in the silkworm organs within the same period (~24 h post infection) between the *L. corymbifera* strain JMRC:FSU:09682 and the *A. fumigatus* strain A1160p+ (2 × 10^4^ vs. 2 × 10^5^ spores).

In addition to the organs, we were also interested in visualizing fungal growth in the hemolymph (blood) of silkworms. However, direct CW staining of the hemolymph on microscope slides often resulted in unspecific staining of dead cells or cell debris. Therefore, we infected silkworms with spores that we had pre-stained with CW. Interestingly, CW apparently not only stained the spore suspension, but the CW staining remained bright enough to follow fungal growth in vivo. This allowed us to easily detect the fungus at different growth stages (spores, swollen spores, germ tubes/short hyphae, and long hyphae) in the hemolymph combining fluorescence and brightfield microscopy. Thus, we were able to monitor the fungal growth in the hemolymph at different time points (8, 16, and 24 h post infection), as demonstrated in Figure S3 using the *A. fumigatus* strain A1160p+. However, for visualization of mature hyphal/mycelial networks in the silkworm organs at the final infection phase, an additional ex vivo CW staining on microscope slides as described above was required. At early time points post infection (8 and 16 h), we could only detect spores and germ tubes/short hyphae in the hemolymph, respectively. While in the end phase of infection (24 h), long hyphae were detected both in the hemolymph and the organs. It seems that the pathogenic fungi grew in the circulation system (hemolymph) of silkworms until the long hyphae accumulated in the organs, which quickly led to the death of silkworms.

### 3.3. Macroscopical and Microscopical Virulence Comparison of Distinct A. fumigatus Strains

After optimization of the silkworm *A. fumigatus* infection model and establishment of the CW staining protocol, we further tested the reliability of our silkworm model by comparing the virulence of previously characterized knockout (KO) mutants of *A. fumigatus* in mice to that in the silkworm model. We first used eight strains from the transcription factor knockout (TFKO) mutant library generated on the basis of the strain A1160p+ [40]. A summary of the comparison is shown in Table 1. Briefly, we infected silkworms either with the indicated TFKO mutant or with the reference strain A1160p+ (*n* = 20 silkworms in each group) and compared the survival of the silkworms post infection. We observed that for TFKO mutants with strongly attenuated (*∆hapB*), attenuated (*∆rfeC* and *∆*AFUB_014000) or unchanged virulence (*∆nctA*) in mouse infections, our silkworm model could faithfully predict these phenotypes, even though various mouse models had been used for individual *A. fumigatus* mutants [40,41]. However, the other four TFKO mutants showed slightly attenuated virulence compared to the WT strain in our silkworm model, which in this case could not reflect the mutant virulence in mouse infections [42,43]. In addition, we also compared seven *A. fumigatus* TFKO mutants that had been previously generated from other WT progenitors and had been validated in various infection models (Table S2). In this case, we tested in silkworms TFKO mutants that had been derived from the A1160p+ parental strain [40] and compared their virulence to the wild type strain. As expected, four TFKO mutants showing attenuated virulence (*∆srbA*, *∆atrR*, *∆hapX,* and *∆acuM*) in our silkworm model displayed similar reduced virulence in mouse models [44,45,46]. The other three TFKO mutants showed slightly attenuated/increased virulence in our silkworm model, which in these cases could not faithfully predict the virulence in mouse models [47,48,49,50,51]. Taken together, for *A. fumigatus* mutants with strongly or moderately attenuated, or unchanged virulence, our silkworm model could faithfully predict the outcome in mouse models. This underlines its usefulness for large-scale preliminary in vivo screening, especially for KO mutants that have previously been overlooked for in vivo characterization in mammalian animal models.

Then, we applied this silkworm model to screen 24 KO mutants of *A. fumigatus* involved in secondary metabolite (SM) biosynthesis, including 12 TFKO mutants and 12 mutants of SM cluster backbone genes (summarized in Table S3 in Supplementary Material). Briefly, we infected silkworms either with the indicated KO mutant or the corresponding WT strain (*n* = 10 silkworms in each group) and compared the survival of the silkworms after infection. Among these 24 mutants, 16 of them showed unchanged virulence compared to the WT strain in our silkworm model, indicating that the corresponding individual genes likely have no impact on fungal virulence. Six mutants (*∆*AFUB_009690, *∆*AFUB_034470, *∆*AFUB_074510, *∆fccR*, *∆tynC*, *∆fmpE*/*∆fsqE*) were slightly attenuated and two mutants (*∆fgaPT2*, *∆*AFUB_034520) slightly enhanced in virulence. In these cases, it might be worth further verifying them in mammalian models of aspergillosis.

In addition to comparing the virulence of distinct *A. fumigatus* strains macroscopically (survival curves), we also asked whether it is feasible to detect the differences microscopically with the help of our CW staining protocol. As a proof-of-concept, we chose three fungal strains for comparison: A1160p+ (WT), *∆hapX* (attenuated virulence), and *∆hapB* (strongly attenuated virulence). We compared the survival (Figure 1c) and fungal burden in silkworm organs at indicated time points (Figure 1d–f). At 26 h post infection, we observed massive hyphal growth only in organs of silkworms infected with A1160p+, while any comparable fungal growth of the *∆hapX* mutant took 50 h. Even 71 h after infection, we observed only minor hyphal growth in the midgut of silkworms infected with *∆hapB*. Thus, the microscopy of fungal burden in organs nicely corresponded with the survival curves of silkworms.

### 3.4. Therapeutic Efficacy and Safety of Conventional Antifungals in Silkworms at 34 °C

Previously, Nakamura et al. [21] had compared the dose-dependent therapeutic efficacy of different conventional antifungals (voriconazole, amphotericin B, micafungin, and caspofungin) in their silkworm *A. fumigatus* infection model (incubation temperature 30 °C, infectious dose 1.5 × 10^6^ spores). As we increased the incubation temperature from 30 to 34 °C, accompanied by a much lower infectious dose, we asked whether our optimized silkworm model would still be suitable for validation of antifungal therapeutic efficacy and safety. In this regard, we chose voriconazole and amphotericin B as representative antifungals to be tested in our silkworm model. The concentration range of voriconazole and amphotericin B, the time points of antifungal treatment, as well as the time points of the evaluation were chosen based on data from Nakamura et al. [21]. Of note, since the fungus grew fast at 34 °C in the silkworms, we further reduced our infectious dose by half to slow down the disease progression concerning infection experiments followed by the antifungal treatment.

**Figure 3:**
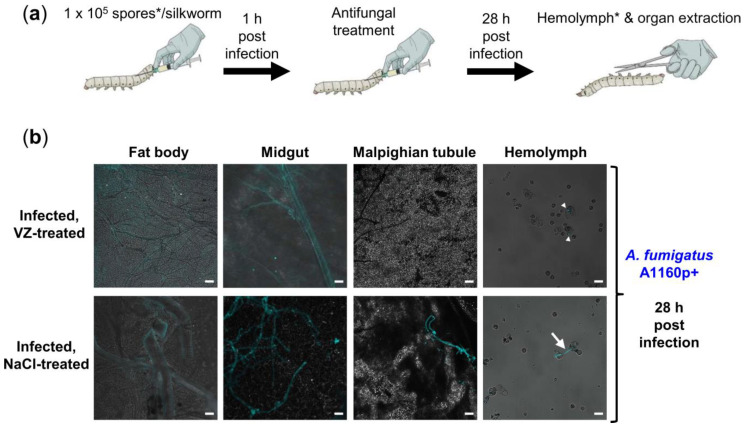
Therapeutic efficacy and safety of voriconazole in *A. fumigatus*-infected silkworms. (**a**) Illustration of experimental setup; treatment of *A. fumigatus*-infected silkworms with voriconazole at doses of either 1, 3.2 or 10 µg/day, respectively, resulted in 100% survival in all treatment groups in comparison to 0% survival of the infected, NaCl-treated controls; * for visualization of the fungus in the hemolymph, calcofluor white pre-stained spores were used for infection; (**b**) merged images (calcofluor white in turquoise + brightfield) show fungal burden in silkworms infected with the *A. fumigatus* A1160p+ strain at 28 h post infection, with or without the voriconazole (VZ) treatment (10 µg/silkworm at 1 h post infection); infected, NaCl-treated control received 20 µL 0.9% NaCl/10% DMSO at the same time point; white arrow heads and white arrow indicate spores and long hypha in the hemolymph, respectively; scale bars represent 20 µm.

Briefly, silkworms infected with 1 × 10^5^ spores of the *A. fumigatus* strain A1160p+ received the antifungal treatment twice at 1 and 24h post infection, respectively. In addition, 46h after infection all silkworms treated with voriconazole (1, 3.2 or 10 µg/day; *n* = 15, 14, or 10 silkworms in each group, respectively) still survived, while all infected controls died without treatment (*n* = 9 silkworms). Moreover, at this time point silkworms receiving the highest dose of voriconazole (10 µg/day) had the same survival rate (100%) as the uninfected and untreated controls, confirming the safety of voriconazole at the tested concentration. Moreover, with the help of our CW staining protocol, we could monitor the antifungal treatment outcome microscopically by comparing the fungal growth in *A. fumigatus*-infected silkworms treated with either 0.9% NaCl or voriconazole (10 µg at 1 h post infection). Hyphal growth was only visible in the hemolymph and organs of silkworms treated with 0.9% NaCl at 28 h post infection (Figure 3b), while those treated with voriconazole only had ungerminated spores in their hemolymph at this time point.

Following the first amphotericin B treatment (1, 3.2 or 10 µg at 1 h post infection), all the treated silkworms survived at 24 h post infection while those left untreated all died (Figure S4b). Following the second amphotericin B treatment (same dose as the first treatment; at 24 h post infection), silkworms showed an antifungal dose-dependent survival at 32 h post infection. The survival rate of silkworms decreased drastically at 46 h post infection. Of note, silkworms receiving the highest dose of amphotericin B (10 µg/day) had a much lower survival rate compared to the control groups, indicating that the silkworms could not tolerate this antifungal at the tested concentration (Figure S4c).

As we had included *L. corymbifera* in this study to prove the general applicability of our CW staining protocol, we further asked whether we could also monitor the antifungal treatment outcome microscopically in *L. corymbifera*-infected silkworms, given that this is a difficult-to-treat fungal pathogen. Of note, voriconazole is generally not used for the treatment of *Lichtheimia* infections in the clinic since it is reportedly inactive [52]. We aimed to compare an active antifungal (amphotericin B) vs. an inactive one (voriconazole) against *L. corymbifera* in vivo.

**Figure 4:**
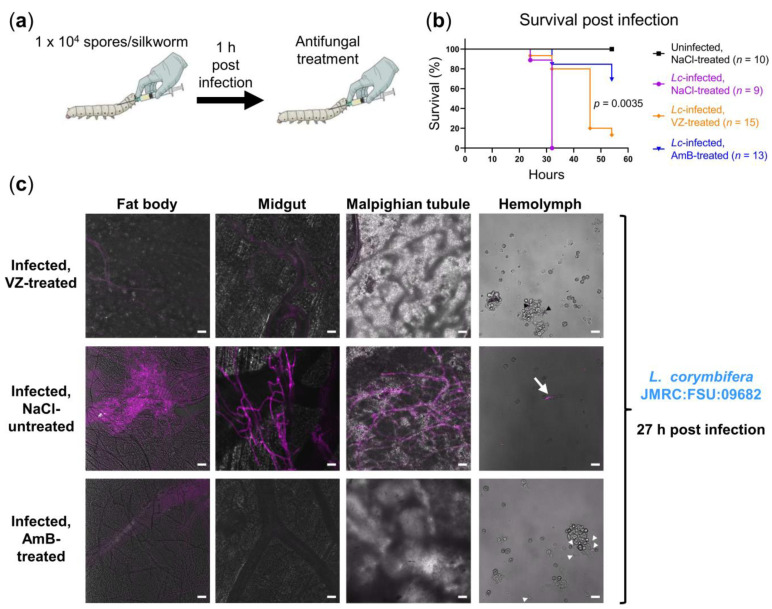
Therapeutic efficacy of voriconazole (VZ) and amphotericin B (AmB) in *L. corymbifera*-infected silkworms. (**a**) Illustration of experimental setup; (**b**) survival of *L. corymbifera*-infected silkworms with or without the antifungal treatment (10 µg voriconazole or amphotericin B at 1 h post infection); uninfected control was first injected with 50 µL NaCl-Tween; NaCl-treated controls received 20 µL 0.9% NaCl/10% DMSO following the first injection or infection; n: Number of silkworms in each group; Kaplan-Meier survival curves were compared using the log-rank test; pairwise comparison was performed between the VZ-treated and AmB-treated groups (*p* = 0.0035); (**c**) merged images (calcofluor white in violet + brightfield) show fungal burden at 27 h post infection; to detect the fungus in hemolymph, calcofluor white pre-stained spores were used for infection; white arrowheads, black arrowheads, and white arrow indicate spores, germinated spores, and long hypha in the hemolymph, respectively; hyphae/mycelia were only visible in the organs of the infected, NaCl-treated control at the indicated time point; scale bars represent 20 µm.

Briefly, we infected silkworms with 1 × 10^4^ spores of *L. corymbifera* and treated them once at 1 h post infection with either 10 µg amphotericin B or voriconazole. Although voriconazole delayed the silkworm death post infection, it could not rescue the insects (Figure 4b). In contrast, 70% of the infected silkworms survived for at least 54h after a single amphotericin B treatment. Figure 4c displays the fungal growth at 27 h post infection in silkworms either treated with 0.9% NaCl, voriconazole or amphotericin B. Corresponding with survival (Figure 4b), invasive fungal growth was only visible in silkworms treated with 0.9% NaCl, while those treated with voriconazole or amphotericin B had only germ tubes/short hyphae or spores in their hemolymph at this time point.

In essence, we established in our proof-of-principle study a simple CW staining protocol for the visualization of fungal burden in filamentous fungus-infected silkworms. This staining protocol enabled us to monitor fungal growth along the disease progression and survival to compare fungal strains with distinct levels of virulence and to validate the therapeutic efficacy of conventional antifungals.

## 4. Discussion

Various invertebrate animal models have proven beneficial as a replacement for mammals to study fungal infections and to validate antifungal drug efficacy at the preliminary stages concerning large-scale experiments. Here, we focused on the silkworm infection model due to the easy handling and low costs, a published genome [53] and large body size facilitating injections and manipulation. In particular, when compared to the larvae of *G. mellonella* and *M. sexta*, both of which have the ability to grow further even after fungal infections into the next developmental stages (larvae → pupae → moths) [15,54], the growth of silkworms is generally inhibited by an incubation temperature higher than 30 °C [35], and the silk moths already lost their ability to fly due to domestication [18]. Thus, the silkworm infection model has a clearly much lower risk of biohazard.

Microscopically monitoring the fungal burden in infected silkworms was a main focus of this study. Starting from a silkworm *A. fumigatus* infection model established by Nakamura et al. [21], we first increased the incubation temperature post infection from 30 to 34 °C to better mimic the situation in humans (37 °C), to speed up the infection experiments, as well as to ease the identification of fungal mutants with attenuated virulence. Of note, although silkworm infection models at 37 °C were established for two yeast pathogens, *C. neoformans* [55] and *T. asahii* [19], we did not choose 37 °C for experiments in this study due to the fact that silkworms could not tolerate this temperature very well (50% survival at 72h). In the case of *C. neoformans*, 37 °C had been necessary for this pathogen to establish infection in the silkworm, and monitoring of survival had been stopped before 70 h [55]. In the case of *T*. *asahii*, the survival of silkworms at 37 °C was monitored for less than 55 h [19]. Unlike these two previous studies, when choosing the incubation temperature, we aimed to find a balance between minimizing thermal stress on silkworms and the rapid growth of *A. fumigatus* in vivo. In our case, incubation at 34 °C accompanied by an infectious dose of 2 × 10^5^ spores was very helpful for the screening of *A. fumigatus* knockout mutants with unaltered virulence, as we could obtain the results within a day after infection. Meanwhile, without bias caused by thermal stress, fungal mutants with attenuated or strongly attenuated virulence could be effectively identified since they generally need more time to progress the infection in silkworms (e.g., 2 and 3 days for the *∆hapX* and *∆hapB* mutants, respectively). In addition, we observed that different clinical isolates of *A. fumigatus* had distinct levels of virulence in silkworms (Figure S2b–e): CEA10 [56] is as virulent as its derivate A1160p+ [36], but more virulent than the genome reference isolate Af293 [57], the D141 strain [58] or the ATCC46645 strain [59,60]. This is in line with previous studies comparing the virulence of CEA10 and Af293 in a non-neutropenic mouse model [61,62], and furthermore emphasizes that the infectious dose used for silkworm experiments must be adjusted individually according to the fungal genetic background.

Following the optimization of silkworm *A. fumigatus* infection model, we asked whether it is feasible to monitor the fungal burden in infected silkworms microscopically, in parallel with monitoring their survival. Applying fluorescent fungal strains for studying fungal burden in infected silkworms has been reported for the dermatophyte *Arthroderma vanbreuseghemii* [23] as well as for the dimorphic fungus *T. asahii* [19]. There, following the disease progression relied on recombinant fungal strains that express fluorescent proteins. In the case of detecting non-fluorescent fungal strains in silkworms, Grocott-Gomori’s methenamine silver stain and periodic acid-Schiff stain, both of which are time-consuming, have been employed for the entomopathogenic fungi *Beauveria bassiana* and *Metarhizium anisopliae*, respectively [24,25].

In this study, we introduced a simple CW staining protocol for visualization of fungal growth in freshly extracted silkworm organs, which drastically eases the detection of fungal pathogens. On the one hand, as demonstrated using *A. fumigatus* and *L. corymbifera*, our staining protocol is applicable for various fungal species, given that CW is generally used for fungal cell wall staining [26]. On the other hand, unlike classical histological staining procedures, our staining protocol does not require fixation, sectioning, and dehydration of samples prior to staining, which minimizes the efforts needed for sample processing. Of note, classical methods such as the Grocott-Gomori’s methenamine silver stain enable high-resolution visualization of both fungal pathogen and host tissue, which is ideal, e.g., for studying host-pathogen interactions.

With the help of CW staining, we could compare *A. fumigatus* strains with distinct levels of virulence along the disease progression in silkworms, both macroscopically and microscopically. Furthermore, we could also monitor the fungal growth at different time points post infection, as well as validate the therapeutic efficacy of antifungals in the hemolymph and organs of silkworms compared to the untreated controls. Of note, in addition to its broad application for fungal cell wall staining, CW is also well known as a stressor that disrupts the cell wall integrity and inhibits fungal growth in a dose-dependent manner [63]. Upon CW pre-staining at the indicated concentration, we observed that spores of *A. fumigatus* or *L. corymbifera* can geminate and grow further in the hemolymph and later also in the organs of silkworms. Nevertheless, for species or mutants that are very sensitive to CW, pre-staining of spore suspension prior to infection may delay or even prevent fungal growth in vivo.

Along the application of our CW staining protocol, we sometimes observed unspecific CW staining of vessels/tissues, especially in the insect’s fat body and less frequently in the midgut. Nonetheless, we had no problem to rapidly distinguish these structures from fungal hyphae via microscopy based on the following points: (1) CW staining of fungal hyphae is generally much brighter than the unspecific staining of vessels/tissues; (2) in the case of *A. fumigatus,* CW not only stains the fungal cell wall, but also the septa within the fungal hyphae (e.g., image of theMalpighian tubule in Figure 1e), which helped us to easily differentiate vessels from hyphae with similar diameters, based on the absence/existence of septa; (3) in the case of *L. corymbifera*, which generally lacks septa within its hyphae [64], distinguishment mostly relied on lateral hyphal branches that were newly established (bud-like structures; e.g., image of the midgut in Figure 2b).

In addition, while validating the reliability of our optimized silkworm *A. fumigatus* infection model by comparing the virulence of previously characterized TFKO mutants in silkworm vs. in mouse models, we observed that for mutants with attenuated, strongly attenuated or unchanged virulence, our silkworm model could faithfully recapitulate the results in mouse models, even though various mouse models were used for individual mutants: Different methods of immunosuppression (leukopenic or non-neutropenic) and different routes of infection (intranasal, intratracheal, intravenous or inhalation) accompanied by diverse infectious doses ranging from 10^3^ to 10^7^ spores/germlings [40,41,44,45,46]. However, for mutants that showed slightly attenuated or increased virulence, our silkworm model was unable to exactly mirror the outcome in mouse models, given that there is no standardized mouse model available [42,43,47,48,49,50,51]. For example, the *∆gliZ* mutant required for gliotoxin biosynthesis showed slightly increased virulence in our silkworm model, while unchanged and attenuated virulence in leukopenic and non-neutropenic mouse models, respectively [49,50]. Although gliotoxin has been shown to inhibit reactive oxygen species (ROS) production of *G. mellonella* hemocytes, identical to its effect on mammalian neutrophils [65], we found controversial data in the literature with respect to ROS production of silkworm hemocytes [66,67] and therefore, could not speculate in the case of *∆gliZ* that results from our silkworm model were closer to that from the leukopenic mouse model. Taken together, mutants showing attenuated or strongly attenuated virulence in our silkworm model are also less virulent or avirulent in mice, while mutants with unaltered virulence are comparable to the WT strain in mice, irrespective of mouse models used. This helps drastically reduce the efforts needed for validation in mice and emphasizes the usefulness of our silkworm model with respect to preliminary large-scale in vivo screening of fungal virulence determinants.

Finally, we tested the applicability of our silkworm models for monitoring the therapeutic efficacy and safety of antifungals in vivo, with voriconazole and amphotericin B as representative drugs. Voriconazole at the tested concentration range (1–10 µg/day) could effectively rescue the silkworms from *A. fumigatus* infection with apparently neglectable toxicity. While amphotericin B (1–10 µg/day) could temporarily slow down the disease progression, silkworms could not tolerate the toxicity of this antifungal, which is in line with clinical observations [68]. Subsequently, we treated silkworms infected with *L. corymbifera*, with the highest dose of antifungals tested in the experiments with *A. fumigatus* but reduced the treatment from twice to only once at 1 h post infection. Of note, liposomal amphotericin B is the first line treatment against *Lichtheimia* infections in the clinic [69]. Since we only used the conventional amphotericin B deoxycholate in this study, which is more toxic than the liposomal formulation [70], we treated the silkworms only once (10 µg/silkworm) to lessen the toxicity of amphotericin B deoxycholate. For comparison, we included also voriconazole (10 µg/silkworm), which is generally not used for treating *Lichtheimia* infections in the clinic due to its inactivity [52]. Interestingly, *L. corymbifera*-infected silkworms treated with voriconazole died slower than the untreated control, nevertheless it could not rescue the silkworms as effectively as the amphotericin B treatment. Taken together, we could validate the therapeutic efficacy and safety of two representative antifungals in two silkworm infection models. This demonstrated the feasibility for verifying novel antifungal agents in silkworms regarding their spectra of activity as well as toxicity, which could help drastically reduce the efforts needed for further validation in mammalian animal models, as ineffective or toxic candidate drugs can already be excluded at this pre-stage.

In summary, in the current study, we introduced a simple CW staining protocol for the visualization of fungal burden in filamentous fungus-infected silkworms to easily monitor the infection progress, to compare fungal strains with distinct virulence, and to validate the therapeutic efficacy of antifungals. Furthermore, we could show that our optimized silkworm *A. fumigatus* infection model is reliable for identification of fungal mutants with attenuated, strongly attenuated or unchanged virulence, which may prove very useful for large-scale in vivo screening to identify fungal virulence determinants. Finally, as demonstrated by using two conventional antifungals, our silkworm infection models proved suitable for validation of novel antifungal agents, particularly helpful to exclude ineffective or toxic candidate drugs at early development stages. Thus, in line with previous studies [14,16,17,18,21] and with the help of CW staining, our research further demonstrates that silkworm is indeed a cost- and time-effective replacement for mammals for preliminary in vivo studies of fungal pathogenicity and early-stage development of novel antifungal agents.

## Figures and Tables

**Table 1:** Virulence of *A. fumigatus* knockout mutants in silkworm vs. in mouse models.

Knockout Gene ID	Generic Name	Virulence in Silkworm Model (Compared to WT *)	Data from Mouse Infection Models (with References)		
			Virulence in Mouse Model (Compared to WT *)	Mouse Model	Infectious Dose
AFUB_030360	*hapB*	Strongly attenuated	Strongly attenuated [41]	Leukopenic	Unknown
AFUB_014000	*-*	Attenuated	Attenuated [41]		
AFUB_026340	*rfeC*	Attenuated	Slightly attenuated [41]		
AFUB_009970	*cbfA*	Slightly attenuated	Unchanged [42]		IN, 1 × 10^5^
AFUB_058240	*nctC*	Slightly attenuated	Avirulent [42]		
AFUB_091020	*fhdA*	Slightly attenuated	Unchanged [42]		
AFUB_008610	*rglT*	Slightly attenuated	Strongly attenuated [43]		
AFUB_029870	*nctA*	Unchanged	Unchanged [40]	Leukopenic	IN, 5 × 10^5^
				Non-neutropenic	IN, 7 × 10^6^

* Wild type (WT) strain in this table means A1160p+; IN: Intranasal infection.

## Data Availability

The data presented in this study are available on request from the corresponding author Y.Y.

## Supplementary Materials

The following are available online at https://www.mdpi.com/2309-608X/7/2/136/s1. Table S1: *Aspergillus fumigatus* strains used in this study; Table S2: Virulence of *A. fumigatus* knockout mutants lacking the same TF-encoding genes in silkworm vs. mouse models; Table S3: Twenty-four *A. fumigatus* KO mutants tested in the silkworm model (regulatory/backbone genes of secondary metabolism); Figure S1: Simplified silkworm rearing protocol (laboratory version); Figure S2: Kaplan-Meier survival curves of silkworms incubated at distinct temperatures without infection or infected with different clinical isolates of *A. fumigatus*; Figure S3: Visualization of fungal burden in hemolymph and organs of *A. fumigatus*-infected silkworms at different time points post infection; Figure S4: Therapeutic efficacy and safety of amphotericin B in *A. fumigatus*-infected silkworms.
