# Supplementary material for: Direct Visualization of Fungal Burden in Filamentous Fungus-Infected Silkworms
Source: J Fungi (Basel). 2021 Feb 13;7(2):136. doi: 10.3390/jof7020136 (PMC7918154; doi:10.3390/jof7020136)
Supplement: Supplementary file 1 [file jof-07-00136-s001.pdf]

Supplementary Material

Table S1. *Aspergillus fumigatus* strains used in this study.

Strain	Progenitor	Description	Reference
ΔakuB^{KU80}	CEA17	*Δku80, pyrG+*	[1]
A1160p+/MFIG001	A1160p-	*Δku80, pyrG+*	[2]
AFUA_1G10550 KO	A1160p+	*ΔcbfA*, hygro^r	[3]
AFUA_5G10620 KO		*ΔnctC*, hygro^r	
AFUA_7G05440 KO		*ΔfhdA*, hygro^r	
AFUA_2G01260 KO		*ΔsrbA*, hygro^r	
AFUA_2G02690 KO		*ΔatrR*, hygro^r	
AFUA_2G14250 KO		*ΔnctA*, hygro^r	
AFUA_5G03920 KO		*ΔhapX*, hygro^r	
AFUA_2G14720 KO		*ΔhapB*, hygro^r	
AFUA_1G14460 KO		ΔAFUA_1G14460, hygro^r	
AFUA_2G10550 KO		*ΔrfeC*, hygro^r	
AFUA_1G09190 KO		*ΔrglT*, hygro^r	
AFUA_3G08520 KO		*ΔrlmA*, hygro^r	
AFUA_1G06900 KO		*ΔcrzA*, hygro^r	
AFUA_2G12330 KO		*ΔacuM*, hygro^r	
AFUA_6G09630 KO		*ΔgliZ*, hygro^r	
AFUA_3G12890 KO		*ΔhasA*, hygro^r	
AFUA_3G14750 KO		ΔAFUA_3G14750, hygro^r	
AFUA_3G15290 KO		ΔAFUA_3G15290, hygro^r	
AFUA_4G14540 KO		*ΔtpcE*, hygro^r	
AFUA_5G10130 KO		ΔAFUA_5G10130, hygro^r	
AFUA_5G10040 KO		ΔAFUA_5G10040, hygro^r	
AFUA_7G00130 KO		*ΔfccR*, hygro^r	

Table S1. *Cont.*

Strain	Progenitor	Description	Reference
AFUA_3G03315 KO	A1160p+	*Δ*AFUA_3G03315, $hygro^r$	[3]
AFUA_6G08550 KO		*Δ*AFUA_6G08550, $hygro^r$	
AFUA_1G10280 KO		*Δ*AFUA_1G10280, $hygro^r$	
AFUA_6G03430 KO		*Δ*AFUA_6G03430, $hygro^r$	
AFUA_2G18040 KO		*ΔfgaPT2*, $hygro^r$	-
AFUA_6G12050 KO		*ΔpesL*, $hygro^r$	
AFUA_6G03480 KO	*ΔakuB^{KU80}*	*ΔfmpE*/*ΔfsqF*, PT^r	
AFUA_6G09660 KO		*ΔgliP*, PT^r	
AFUA_8G00540 KO		*ΔpsoA*, PT^r	
AFUA_8G00370 KO		*ΔfmaB*, PT^r	
AFUA_8G00420 KO		*ΔfumR*/*ΔfapR*, PT^r	
AFUA_4G14560 KO		*ΔtynC*, $hygro^r$	
AFUA_7G00160 KO		*ΔfccA*, PT^r	
AFUA_7G00170 KO		*ΔfccD*, PT^r	
AFUA_5G02330 KO		*Δaspf1*, PT^r	
AFUA_8G00170 KO		*ΔftmA*, PT^r	
AFUA_3G14700 KO		*Δ*AFUA_3G14700, $hygro^r$	

KO: Knockout (Δ) mutant; $hygro^r$: Hygromycin resistance; PT^r: Pyrithiamine resistance.

Table S2. Virulence of *A. fumigatus* knockout mutants lacking the same TF-encoding genes in silkworm *vs.* in mouse models.

Knockout gene ID		Generic name	Virulence in silkworm model (compared to WT*)	Data from mouse infection models (with references)			
				Virulence in mouse model (compared to WT)	Fungal WT	Mouse model	Infectious dose (spores)
AFUA_2G01260	AFUB_018340	*srbA*	Attenuated	Strongly attenuated[4]	AfS35	Leukopenic	IT, 3x10^{5}
AFUA_2G02690	AFUB_019790	*atrR*	Attenuated	Strongly attenuated[4]	AfS35	Leukopenic	IT, 3x10^{5}
					Af293		IT, 2.5x10^{7}
AFUA_5G03920	AFUB_052420	*hapX*	Attenuated	Attenuated[5]	ATCC46645	Leukopenic	IN, 6x10^{4}
				Avirulent[5]		Non-neutropenic	
AFUA_2G12330	AFUB_027990	*acuM*	Attenuated	Attenuated[6]	Af293	Leukopenic	IV, 3x10^{3} germlings
						Non-neutropenic	IH
AFUA_3G08520	AFUB_040580	*rlmA*	Slightly attenuated	Strongly attenuated[7]	Δaku^{KU80}	Leukopenic	IN, 2.5x10^{6}
AFUA_1G06900	AFUB_007280	*crzA*	Slightly attenuated	Avirulent[8]	Af293	Leukopenic	IH
				Strongly attenuated[9]	$\Delta akuB^{KU80}$		IN 2.5-5.0x10^{5}
AFUA_6G09630	AFUB_075680	*gliZ*	Slightly increased	Unchanged[10]	Af293.1	Leukopenic	IN, 2.5x10^{5}
				Attenuated[11]	B-5233	Non-neutropenic	IN, 5x10^{6}

*Wild type (WT) strain in this study means A1160p+ (derived from CEA10); AfS35 and Δaku^{KU80} are derived from D141 and CEA10, respectively; IT: Intratracheal infection; IN: Intranasal infection; IV: Intravenous infection; IH: Inhalation.

Table S3. Twenty-four *A. fumigatus* KO mutants tested in the silkworm model (regulatory/backbone genes of secondary metabolism).

Knockout (KO) gene ID		Generic name	SM cluster product	Virulence in silkworm model (compared to WT)	Fungal WT
AFUA_1G10280	AFUB_009690	- (TF)	Unknown	Slightly attenuated	A1160p+
AFUA_3G14750	AFUB_034470	- (TF)	Unknown		
AFUA_6G08550	AFUB_074510	- (TF)	Unknown		
AFUA_7G00130	AFUB_086680	*fccR* (TF)	Fumicycline		
AFUA_4G14560	AFUB_071800	*tynC* (PKS)	Trypacidin		$\Delta akuB^{KU80}$
AFUA_6G03480	AFUB_094810	*fmpE/fsqF* (NRPS-like)	Fumipyrrole/Fumisoquin		
AFUA_3G12890	AFUB_036300	*hasA* (TF)	Hexadehydroastechrome	Unchanged	A1160p+
AFUA_3G15290	AFUB_033930	- (TF)	Unknown		
AFUA_4G14540	AFUB_071780	*tpcE* (TF)	Trypacidin		
AFUA_5G10130	AFUB_057730	- (TF)	Unknown		
AFUA_5G10040	AFUB_057630	- (TF)	Unknown		
AFUA_6G03430	AFUB_094860	*fmpR* (TF)	Fumipyrrole/Fumisoquin		
AFUA_6G12050	AFUB_078040	*pesL* (NRPS)	Unknown		
AFUA_3G03315	AFUB_044930	- (TF)	Siderophore		
AFUA_6G09660	AFUB_075710	*gliP* (NRPS)	Gliotoxin		$\Delta akuB^{KU80}$
AFUA_8G00540	AFUB_086030	*psoA* (PKS-NRPS)	Pseurotin		
AFUA_8G00370	AFUB_086200	*fmaB* (PKS)	Fumagillin		
AFUA_8G00420	AFUB_086150	*fumR/fapR* (TF)	Fumagillin/Pseurotin		
AFUA_7G00160	AFUB_086700	*fccA* (PKS)	Fumicycline		
AFUA_7G00170	AFUB_086710	*fccD* (DMAT)	Fumicycline		
AFUA_5G02330	AFUB_050860	*aspf1*	AspF1		
AFUA_8G00170	AFUB_086360	*ftmA* (NRPS)	Fumitremorgin		
AFUA_2G18040	AFUB_033730	*fgaPT2* (DMAT)	Fumigaclavin	Slightly increased	A1160p+
AFUA_3G14700	AFUB_034520	- (PKS)	Unknown		$\Delta akuB^{KU80}$

SM: secondary metabolite; TF: Transcription factor-encoding gene; PKS: Polyketide synthase-encoding gene; NRPS: Nonribosomal peptide synthetase-encoding gene; DMAT: Dimethylallyl tryptophan synthase-encoding gene.

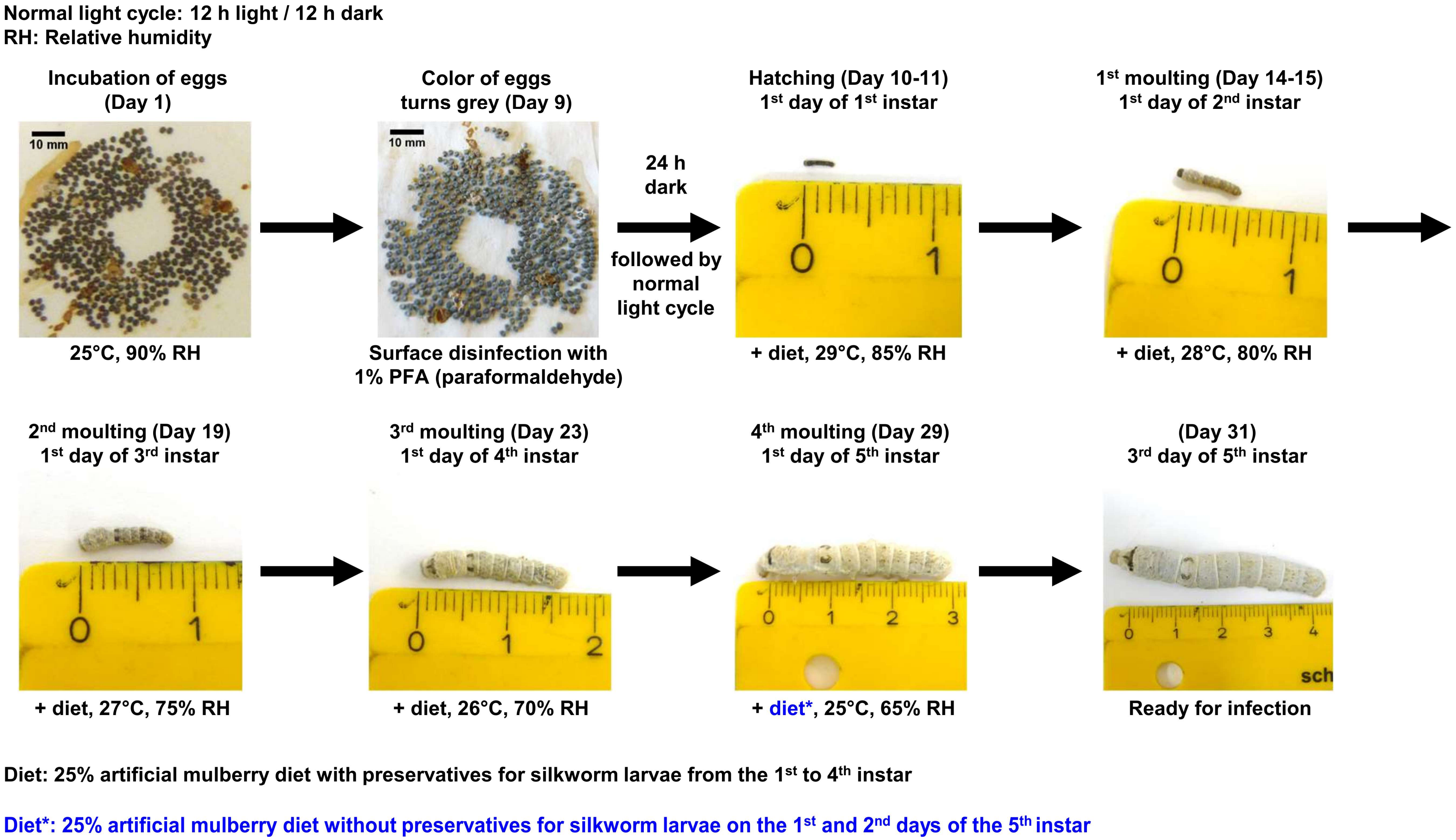

Figure S1. Simplified silkworm rearing protocol (laboratory version).

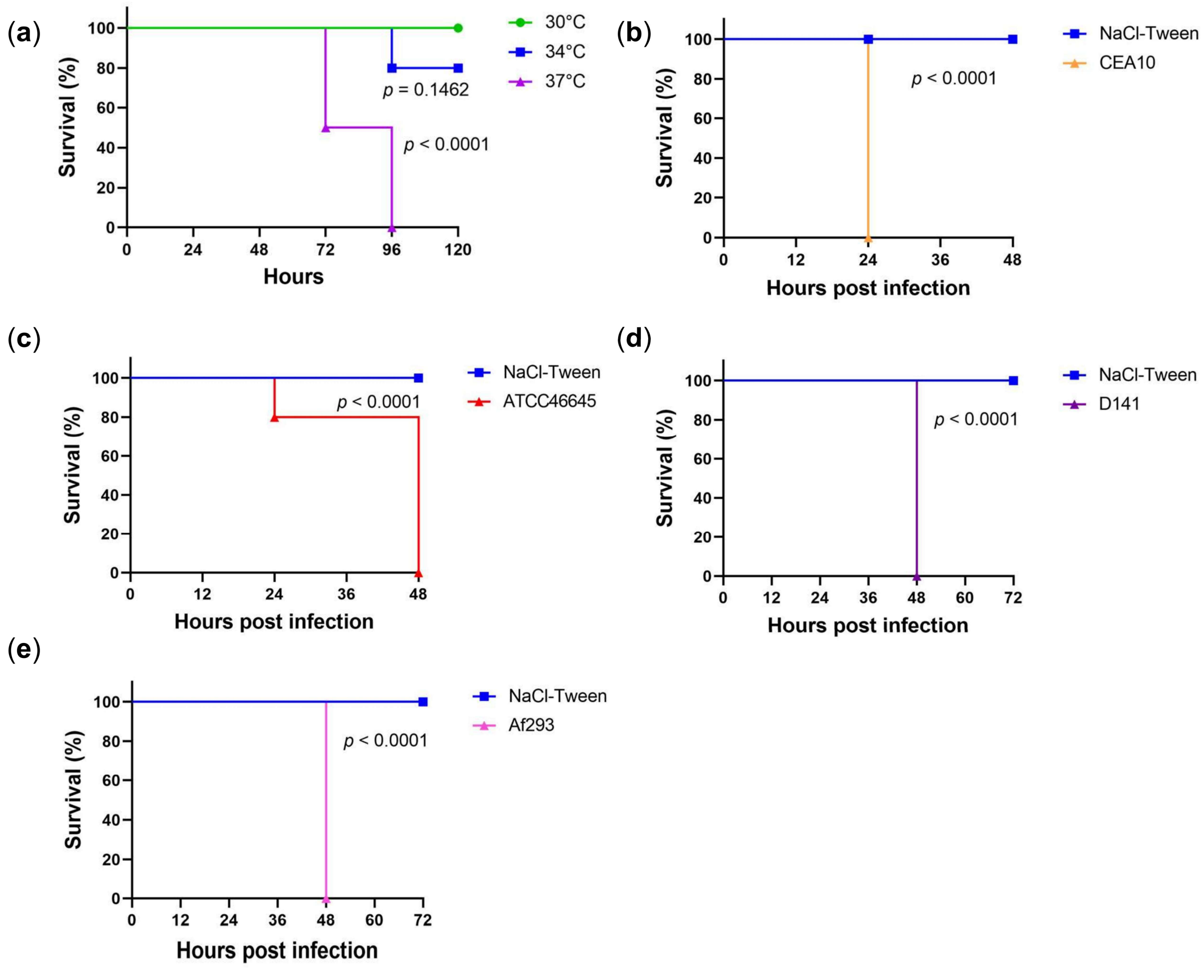

Figure S2. Kaplan-Meier survival curves of silkworms incubated at distinct temperatures without infection or infected with different clinical isolates of *A. fumigatus*. (**a**) Silkworms were fasted during the incubation at indicated temperatures (10 silkworms/group); Kaplan-Meier survival curves were compared using the log-rank test; pairwise comparisons were performed between 30°C and 34°C (p = 0.1462), as well as between 30°C and 37°C (p < 0.0001); (**b**)-(**e**) Silkworms were infected with indicated clinical isolates of *A. fumigatus* (10 silkworms/group); silkworms of the control group (n=10) were injected with 50 µl NaCl-Tween; Kaplan-Meier survival curves were compared using the log-rank test; pairwise comparisons were performed between silkworms infected with the indicated fungal isolate and the control group.

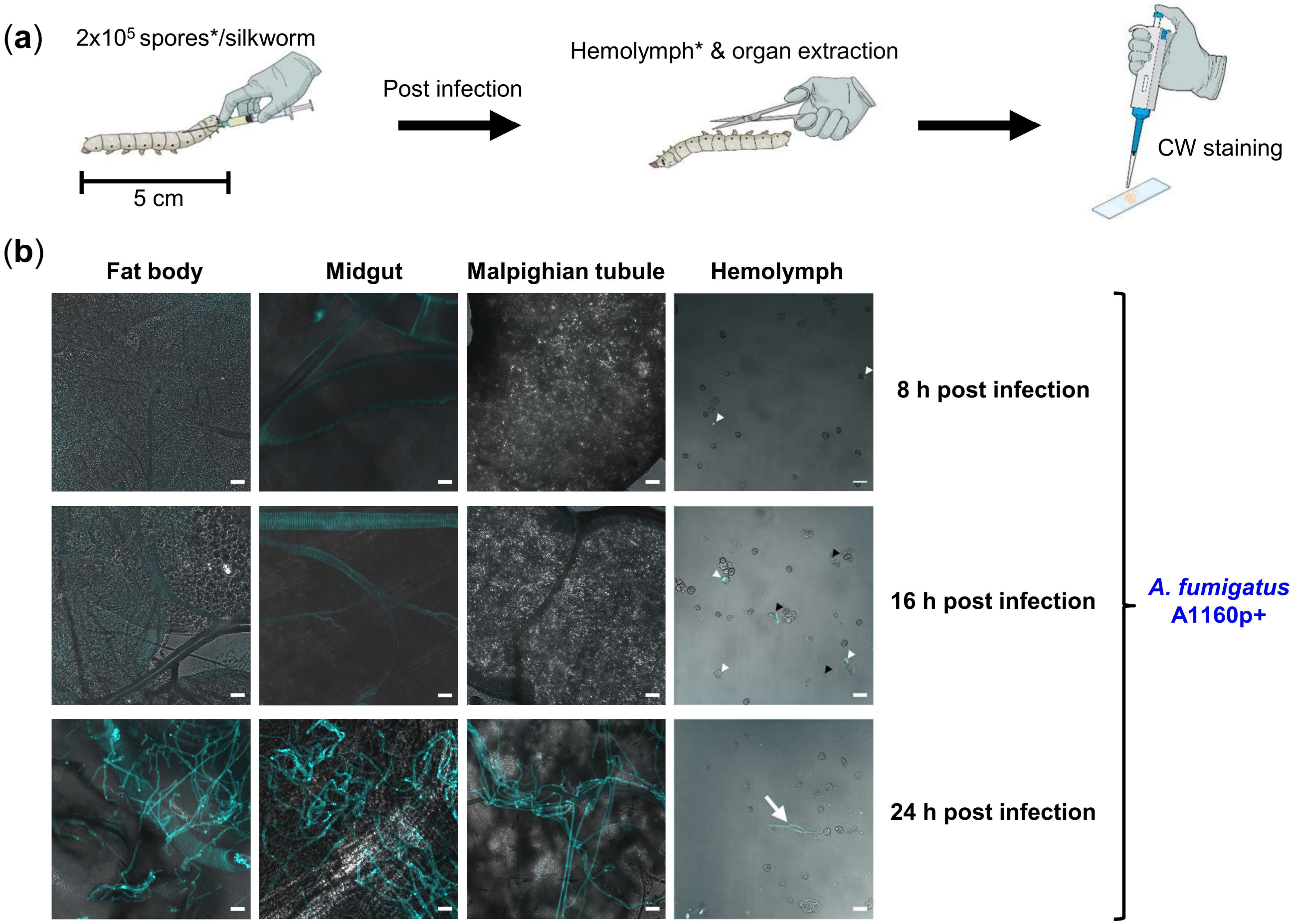

Figure S3. Visualization of fungal burden in hemolymph and organs of *A. fumigatus*-infected silkworms at different time points post infection. (**a**) Illustration of experimental setup; CW: calcofluor white; *for visualization of the fungus in the hemolymph, CW pre-stained spores were used for infection; (**b**) Merged images (calcofluor white in turquoise + brightfield) show fungal growth along the disease progression in the hemolymph: spores (white arrow heads) at 8 h, germ tubes/short hyphae (black arrow heads) at 16 h, and long hyphae (white arrow) at 24 h post infection; hyphae were only detected in the organs at 24 h post infection; scale bars represent 20 μm.

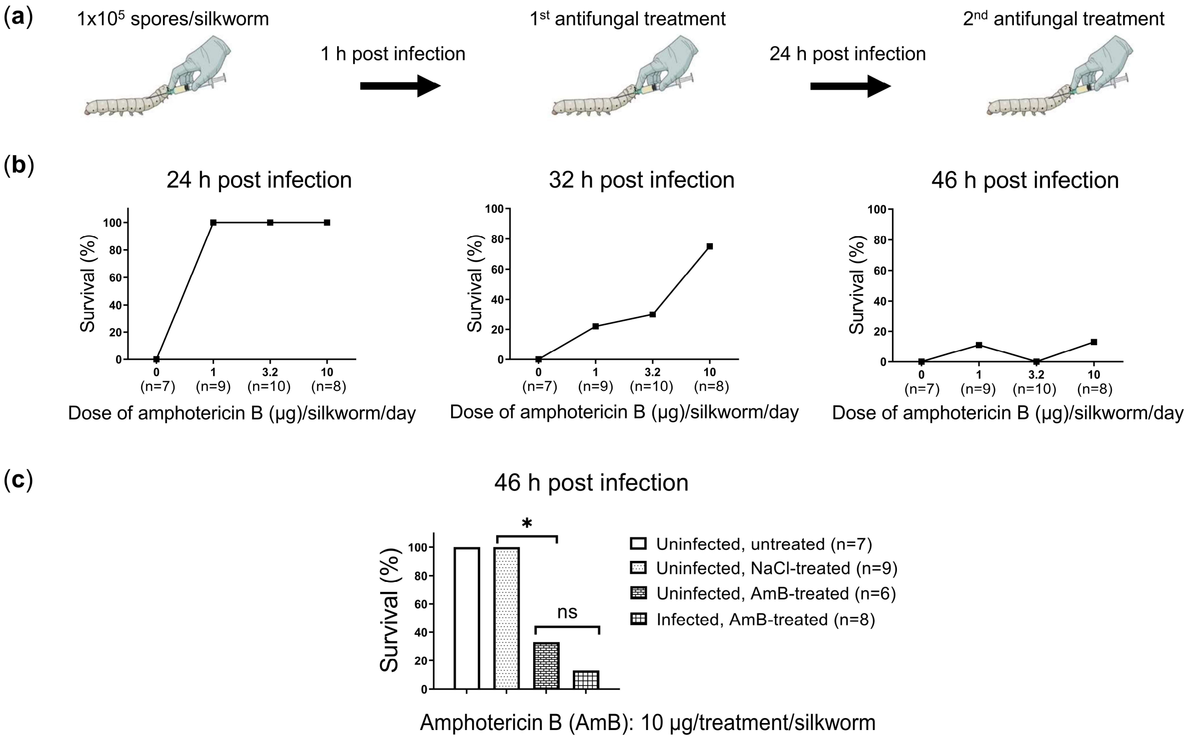

Figure S4. Therapeutic efficacy and safety of amphotericin B in *A. fumigatus*-infected silkworms. (**a**) Illustration of experimental setup, silkworms were reared at 34°C without feeding post infection; (**b**) Dose-dependent therapeutic efficacy of amphotericin B in terms of silkworm survival rate at 24 h, 32 h, and 46 h post infection; n: number of silkworms in each group; (**c**) Severe toxicity of amphotericin B (10 µg/day) in silkworms; uninfected controls were first injected with 50 µl NaCl-Tween; NaCl-treated control was injected with 40 µl 0.9% NaCl at time point 1 h and 24 h following the first injection; statistical analysis of the Kaplan-Meier survival curves of indicated groups (log-rank test; pairwise comparison) was integrated in this diagram, * means p-value < 0.05; ns means not significant (p > 0.05).

REFERENCES

1. da Silva Ferreira, M.E.; Kress, M.R.; Savoldi, M.; Goldman, M.H.; Härtl, A.; Heinekamp, T.; Brakhage, A.A.; Goldman, G.H. The *akuB*^{KU80} mutant deficient for nonhomologous end joining is a powerful tool for analyzing pathogenicity in *Aspergillus fumigatus*. *Eukaryot. Cell*. **2006**. *5*, 207-211. DOI: 10.1128/ec.5.1.207-211.2006
2. Fraczek, M.G.; Bromley, M.; Buied, A.; Moore, C.B.; Rajendran, R.; Rautemaa, R.; Ramage, G.; Denning, D.W.; Bowyer, P. The *cdr1B* efflux transporter is associated with non-*cyp51a*-mediated itraconazole resistance in *Aspergillus fumigatus*. *J. Antimicrob. Chemother*. **2013**. *68*, 1486-1496. DOI: 10.1093/jac/dkt075
3. Furukawa, T.; van Rhijn, N.; Fraczek, M.; Gsaller, F.; Davies, E.; Carr, P.; Gago, S.; Fortune-Grant, R.; Rahman, S.; Gilsenan, J.M.; Houlder, E.; Kowalski, C.H.; Raj, S.; Paul, S.; Cook, P.; Parker, J.E.; Kelly, S.; Cramer, R.A.; Latgé, J.P.; Moye-Rowley, S.; Bignell, E.; Bowyer, P.; Bromley, M.J. The negative cofactor 2 complex is a key regulator of drug resistance in *Aspergillus fumigatus*. *Nat. Commun*. **2020**. *11*, 427. DOI: 10.1038/s41467-019-14191-1
4. Hagiwara, D.; Miura, D.; Shimizu, K.; Paul, S.; Ohba, A.; Gonoi, T.; Watanabe, A.; Kamei, K.; Shintani, T.; Moye-Rowley, W.S.; Kawamoto, S.; Gomi, K. A novel Zn2-Cys6 transcription factor AtrR plays a key role in an azole resistance mechanism of *Aspergillus fumigatus* by co-regulating *cyp51A* and *cdr1B* expressions. *PLoS Pathog*. **2017**. *13*, e1006096. DOI: 10.1371/journal.ppat.1006096
5. Schrettl, M.; Beckmann, N.; Varga, J.; Heinekamp, T.; Jacobsen, I.D.; Jöchl, C.; Moussa, T.A.; Wang, S.; Gsaller, F.; Blatzer, M.; Werner, E.R.; Nierman, W.C.; Brakhage, A.A.; Haas, H. HapX-mediated adaption to iron starvation is crucial for virulence of *Aspergillus fumigatus*. *PLoS Pathog*. **2010**. *6*, e1001124. DOI: 10.1371/journal.ppat.1001124
6. Liu, H.; Gravelat, F.N.; Chiang, L.Y.; Chen, D.; Vanier, G.; Ejzykowicz, D.E.; Ibrahim, A.S.; Nierman, W.C.; Sheppard, D.C.; Filler, S.G. *Aspergillus fumigatus* AcuM regulates both iron acquisition and gluconeogenesis. *Mol. Microbiol*. **2010**. *78*, 1038-1054. DOI: 10.1111/j.1365-2958.2010.07389.x
7. Rocha, M.C.; Fabri, J.H.; Franco de Godoy, K.; Alves de Castro, P.; Hori, J.I.; Ferreira da Cunha, A.; Arentshorst, M.; Ram, A.F.; van den Hondel, C.A.; Goldman, G.H.; Malavazi, I. *Aspergillus fumigatus* MADS-box transcription factor *rlmA* is required for regulation of the cell wall integrity and virulence. *G3 (Bethesda)*. **2016**. *6*, 2983-3002. DOI: 10.1534/g3.116.031112
8. Cramer, R.A.J.; Perfect, B.Z.; Pinchai, N.; Park, S.; Perlin, D.S.; Asfaw, Y.G.; Heitman, J.; Perfect, J.R.; Steinbach, W.J. Calcineurin target CrzA regulates conidial germination, hyphal growth, and pathogenesis of *Aspergillus fumigatus*. *Eukaryot. Cell*. **2008**. *7*, 1085-1097. DOI: 10.1128/ec.00086-08
9. Soriani, F.M.; Malavazi, I.; da Silva Ferreira, M.E.; Savoldi, M.; Von Zeska Kress, M.R.; de Souza Goldman, M.H.; Loss, O.; Bignell, E.; Goldman, G.H. Functional characterization of the *Aspergillus fumigatus* CRZ1 homologue, CrzA. *Mol. Microbiol*. **2008**. *67*, 1274-1291. DOI: 10.1111/j.1365-2958.2008.06122.x
10. Bok, J.W.; Chung, D.; Balajee, S.A.; Marr, K.A.; Andes, D.; Nielsen, K.F.; Frisvad, J.C.; Kirby, K.A.; Keller, N.P. GliZ, a transcriptional regulator of gliotoxin biosynthesis, contributes to *Aspergillus fumigatus* virulence. *Infect. Immun*. **2006**. *74*, 6761-6768. DOI: 10.1128/iai.00780-06
11. Sugui, J.A.; Pardo, J.; Chang, Y.C.; Zarember, K.A.; Nardone, G.; Galvez, E.M.; Müllbacher, A.; Gallin, J.I.; Simon, M.M.; Kwon-Chung, K.J. Gliotoxin is a virulence factor of *Aspergillus fumigatus*: *gliP* deletion attenuates virulence in mice immunosuppressed with hydrocortisone. *Eukaryot. Cell*. **2007**. *6*, 1562-1569. DOI: 10.1128/ec.00141-07
